# Toll-Like Receptor 2 (TLR2) and TLR4 Mediate the IgA Immune Response Induced by Mycoplasma hyopneumoniae

**DOI:** 10.1128/IAI.00697-19

**Published:** 2019-12-17

**Authors:** Xia Li, Yun-ke Zhang, Bao Yin, Jing-bo Liang, Fei Jiang, Wen-xue Wu

**Affiliations:** a Key Laboratory of Animal Epidemiology and Zoonosis, College of Veterinary Medicine, China Agricultural University, Beijing, China; b National Feed Drug Reference Laboratories, Feed Research Institute, Chinese Academy of Agricultural Sciences, Beijing, China; c China Animal Disease Control Center (CADC), Beijing, China; Washington State University

**Keywords:** *Mycoplasma hyopneumoniae*, IgA, DC, B cells, TLR

## Abstract

IgA plays an important role in mucosal immunity against infectious pathogens; however, the molecular mechanism of IgA secretion in response to infection remains largely unknown, particularly in Mycoplasma spp. In this study, we found that the levels of IgA in the peripheral blood serum, bronchoalveolar lavage fluid, nasal mucosa, trachea, hilar lymph nodes, and lung tissues of pigs increased significantly after infection with Mycoplasma hyopneumoniae. Furthermore, IgA and CD11c were detected in the lungs and hilar lymph nodes by immunohistochemical analysis, and colocalization of these two markers indicates that CD11c^+^ cells play an important role in IgA mucosal immunity induced by M. hyopneumoniae. To investigate the regulatory mechanism of IgA, we separated mouse dendritic cells (DCs) from different tissues and mouse macrophages from the lungs and then cultured mouse B cells together with either DCs or macrophages *in vitro*. In the mouse lung-DC/B (LDC/B) cell coculture, IgA secretion was increased significantly after the addition of whole-cell lysates of M. hyopneumoniae. The expression of both Toll-like receptor 2 (TLR2) and TLR4 was also upregulated, as determined by mRNA and protein expression analyses, whereas no obvious change in the expression of TLR3 and TLR7 was detected. Moreover, the IgA level decreased to the same as the control group when TLR2 or TLR4 was inhibited instead of TLR8 or TLR7/9. In conclusion, M. hyopneumoniae can stimulate the response of IgA through TLR2 and TLR4 in a mouse LDC/B cell coculture model, and the coculture model is an ideal tool for studying the IgA response mechanism, particularly that with *Mycoplasma* spp.

## Abstract (precis)

IgA plays an important role in mucosal immunity against infectious pathogens; however, the molecular mechanism of IgA secretion in response to infection remains largely unknown, particularly in Mycoplasma spp. In this study, we found that the levels of IgA in the peripheral blood serum, bronchoalveolar lavage fluid, nasal mucosa, trachea, hilar lymph nodes, and lung tissues of pigs increased significantly after infection with Mycoplasma hyopneumoniae.

## INTRODUCTION

*Mycoplasma hyopneumoniae* is one of the primary pathogens of porcine enzootic pneumonia. It usually can destroy the respiratory epithelium and predispose pigs to secondary infection with other bacteria and viruses, and it has caused huge economic loss in the pig industry worldwide (1, 2). As reported in many pathogenesis studies, M. hyopneumoniae adheres to the respiratory epithelium via adhesion factors such as p97 (3), p102 (4), and p146 (5) after invading the airway of pigs. Some lipid-associated membrane proteins have been proven to be able to induce cell apoptosis and promote the production of reactive oxygen species (ROS) (6), and the toxic metabolite (hydrogen peroxide) is an effective virulence factor of mycoplasmas, including M. hyopneumoniae (7, 8). Recently, a double-protein system consisting of Ig-binding protein and Ig degradation protein was found in Mycoplasma mycoides subsp. capri, which could capture and cleave immunoglobulin G and is believed to play a potentially key role in the immune evasion by *Mycoplasma* spp. After genetic comparison, the researchers found that M. hyopneumoniae also contains homologous genes of the system (9). In response to M. hyopneumoniae infection, pigs usually developed higher levels of immunoglobulin, and IgA response was detected earlier than serum IgG response for M. hyopneumoniae (10). A high level of IgA immune responses has been also reported in pigs immunized with M. hyopneumoniae (11–13) or a chimeric protein containing M. hyopneumoniae antigens (14). It is believed that M. hyopneumoniae induces intense mucosal immune responses and that long-lasting IgA may provide indispensable immune protection for the organism. However, there are few studies about the molecular mechanism by which M. hyopneumoniae promotes such strong mucosal immunity characterized by the increase in IgA.

As the principal mucosal antibody class, IgA is synthesized by local plasma cells and serves as the first line of immune defense against pathogenic microorganisms on the mucosal surface. IgA is synthesized by local plasma cells only after class-switch recombination (CSR) of the Ig heavy chains (15). Various cytokines, costimulators, and cells have been identified that can regulate the CSR program, including T cells and dendritic cells (DCs). IgA class switching can occur in both T cell-dependent and -independent pathways (16, 17). Intestinal DCs can retain small numbers of live commensals for several days and selectively induce IgA (18, 19), while lung DCs have been shown to induce both T cell-dependent and -independent IgA responses through the release of several IgA-inducing factors, including B cell-activating factor (BAFF; also known as BLyS), a proliferation-inducing ligand (APRIL), transforming growth factor beta 1 (TGF-β1), interleukin 6 (IL-6), and IL-10 (20, 21). Using a DC/B cell coculture model stimulated with lipopolysaccharide (LPS), DCs were found to be able to increase B cell proliferation and regulate IgA production, and B cells could direct the maturation and function of DCs (22–24).

Previous reports showed that the microbiota imprints lung DCs with the capacity to induce IgA CSR dependent on MyD88 and TIR-domain-containing adapter-inducing interferon-β (TRIF), which are junction molecules of the Toll-like receptor regulation pathway (25). Studies have reported the IgA response targeting lipoprotein Z (LppZ) of Mycobacterium tuberculosis (26) and antigen-specific secretory IgA responses upon intranasal immunization with pneumococcal surface protein A (PspA) plus cholera toxin (CT) (26–28). *Mycoplasma* spp. are characterized by a lack of a cell wall, and these organisms possess abundant lipoproteins on the surface of the cell membrane. Macrophage-activating lipopeptide 2 (MALP-2) from Mycoplasma fermentas confers host immune activation through Toll-like receptor 2 (TLR2) (29), while triacylated lipoproteins derived from Mycoplasma pneumoniae and Mycoplasma genitalium can activate nuclear factor-κB (NF-κB) through TLR1 and TLR2 (30, 31), causing a strong mucosal immune response. Furthermore, reports have shown that immunization of guinea pigs with chimeric recombinant protein HP14/30 from M. pneumoniae induces high, sustained IgA levels in respiratory tract samples, such as bronchoalveolar lavage fluid (BALF) and nasal and throat lavage samples (32). An increasing number of *Mycoplasma* components has been reported to elicit IgA immune activation; however, the detailed pathways and mechanisms involved remain unclear.

In this study, we established infection in pigs with M. hyopneumoniae, analyzed the changes of antibodies in pig serum, nasal swabs, and BALF at an early stage of infection, and detected the immune cells involved by immunohistochemistry. Furthermore, we established a mouse DC/B cell coculture model to evaluate the IgA immune response stimulated by M. hyopneumoniae and the mechanism involved.

## RESULTS

### IgA increased significantly at the early stage of M. hyopneumoniae infection.

**FIG 1:**
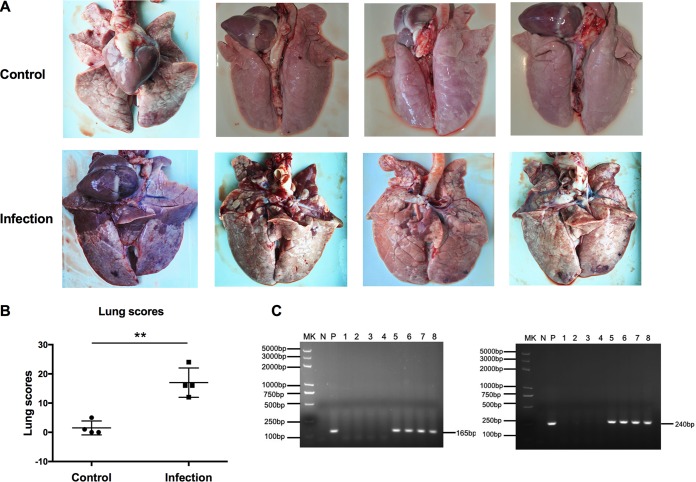
Lesion scores and pathogen detections of lungs from pigs infected with M. hyopneumoniae. (A) Gross observation of the lungs 20 days after M. hyopneumoniae infection. (B) Statistical analysis of lung scores in the infection and control groups. *t* tests of two-tailed analysis were performed to detect significance; **, *P* < 0.01. (C) Detection of M. hyopneumoniae conserved genes in the lung tissues from the infection and control groups by PCR. The gene used in the PCRs for detecting M. hyopneumoniae was one encoding a conserved hypothetical protein and is named *mhp165* (GenBank accession no. AE017332, M. hyopneumoniae strain 232 complete genome; bp 195124 to 201267); the target gene fragments we chose in this paper were bp 199034 to 199198 (MHP165, left) and bp 199131 to 199370 (MHP240, right). Lane MK, DNA marker; lane N, PCR negative control; lane P, M. hyopneumoniae PCR positive control; lanes 1 to 4, control group; lanes 5 to 8, infection group.

**FIG 2:**
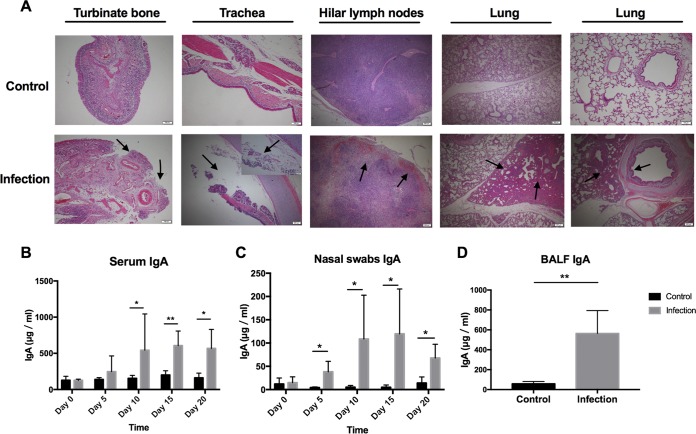
Hematoxylin and eosin stains of various tissue sections and IgA levels in different samples from the infected and control pigs. (A) Hematoxylin and eosin staining of turbinate bone, trachea, hilar lymph nodes, and lung abscissions from M. hyopneumoniae-infected and control pigs. The black arrow indicates the pathological areas. The scale bar is shown in the lower right corner of each image. (B and C) Serum IgA and nasal swab IgA were detected every 5 days by ELISA. (D) BALF IgA concentrations in 300 ml lavage fluid, as detected by ELISA. Each histogram represents the average IgA level of four pigs in the infected group (gray) or the control group (black). A Mann-Whitney test was performed to analyze the statistical significance of the serum IgA levels on day 10 and day 15 between the infected group and the control group since the data did not fulfill the criteria of normality. Analysis of significance of the other data was performed using two-tailed *t* tests. *, *P* < 0.05; **, *P* < 0.01.

M. hyopneumoniae-negative pigs (see Fig. S1A in the supplemental material) were randomly divided into two groups, the M. hyopneumoniae infection group and the control group. The infected pigs showed mild symptoms, such as cough, but the diet and mental state seemed to be normal. After 20 days of M. hyopneumoniae infection, the pigs were euthanized for pathological dissection. The heart lobe, tip lobe, and middle lobe of the lung all showed pulmonary changes and carnification (Fig. 1A). The lung lesion scores were significantly higher than those of the control group (Fig. 1B). Pulmonary lymph nodes and mediastinal lymph nodes were hemorrhagic and enlarged. A mass of DCs, macrophages, neutrophils, and lymphocytes accumulated in the alveolar spaces. PCR analysis of the conserved genes of M. hyopneumoniae in the lesioned lung tissues of the infection group showed positive results (Fig. 1C). The gene used in the PCRs for detecting M. hyopneumoniae encodes a conserved hypothetical protein and is named *mhp165* (GenBank accession no. AE017332, M. hyopneumoniae strain 232 complete genome; bp 195124 to 201267) (33); the target gene fragments we chose in this paper were bp 199131 to 199370 (MHP240) and bp 199034 to 199198 (MHP165). Hematoxylin and eosin staining revealed different cellular states between the control group and the infected group. After M. hyopneumoniae infection, neutrophils infiltrated the nasal and trachea mucosal abscission, the hilar lymph nodes became hemorrhagic, and the lungs were characterized by interstitial pneumonia and hyperplasia of type II pneumocytes. Furthermore, this pneumonia involved bronchovascular thickening, centrilobular nodules, ground-glass attenuation, and air space consolidation (Fig. 2A). The above-mentioned data indicated that we had successfully established infection in pigs with M. hyopneumoniae.

To detect the changes of IgA contents, peripheral blood and nasal swabs were collected every 5 days, and BALF was collected on day 20 after infection with M. hyopneumoniae. Serum IgA levels rose significantly at day 10 (3.5-fold higher than the control group), day 15 (3.3-fold higher than the control group), and day 20 (3.5-fold higher than the control group) (Fig. 2B). The IgA levels in nasal swabs increased significantly at day 5 (8.7-fold higher than the control group), day 10 (21-fold higher than the control group), day 15 (24-fold higher than the control group), and day 20 (4.8-fold higher than the control group) (Fig. 2C). Moreover, IgA levels in BALF increased 9.8-fold compared to the control group (Fig. 2D). However, there was no significant difference in the contents of IgG and IgM in peripheral blood between the two groups (Fig. S1B), and although the amounts of IgG and IgM in the nasal swabs of the infected group increased from day 15 compared with the control group, there was no significant difference except for the IgM amount on day 10, which indicated that obvious mucosal immune responses characterized by increased IgA were induced by M. hyopneumoniae infection.

### IgA and CD11c colocalized in lesion tissues.

**FIG 3:**
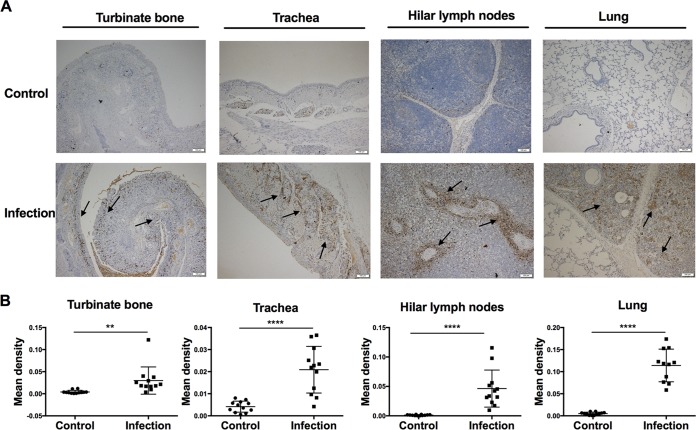
Detection of IgA in various tissue sections from M. hyopneumoniae-infected and control pigs by immunohistochemical staining. (A) Immunohistochemical stains of turbinate bone, trachea, hilar lymph nodes, and lung. The black arrow indicates IgA-positive fields. The scale bar is shown in the lower right corner of each image. (B) Statistical analysis of IgA in turbinate, trachea, lymph node, and lung tissue sections stained by immunohistochemical method. Integrated option density (IOD) and area were measured using software Image-Pro Plus to assess IgA levels quantitatively, and each dot represents the IgA-positive signal mean density (IOD/area) in one section. For each tissue, IgA-positive signal mean densities from three sections were calculated for each of the four pigs in the infected group or control group. In total, 12 data points for each tissue were tested to analyze the significance between the infected group and the control group with two-tailed *t* tests. **, *P* < 0.01; ****, *P* < 0.0001.

To determine the response in the mucosal immune system after infection, we measured IgA^+^ B cell numbers in M. hyopneumoniae-infected tissues by an immunohistochemical method. We found intensive distribution of IgA in the immunohistochemical stained sections of turbinate bone and trachea, especially around the trabecula of the lymph nodes and peritrabecular sinuses. We also found more IgA distributed diffusely in the cilia and the lung tissues with pathological changes (Fig. 3A). Moreover, we found a higher positive rate of IgA in the air space consolidated lung lesions in the infection group than the control group. The differences in the amounts of IgA in turbinate bones, trachea, hilar lymph nodes, and lung tissues are statistically significant between the two groups (Fig. 3B).

**FIG 4:**
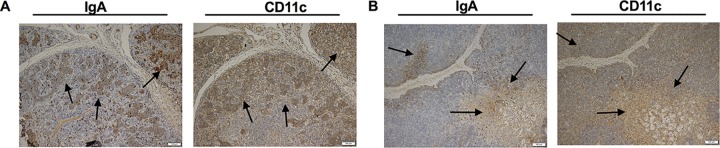
Detection of IgA and CD11c in the M. hyopneumoniae-infected pigs by immunohistochemical staining. (A) Detection of IgA and CD11c in lung tissue by the continuous slices. (B) Detection of IgA and CD11c in hilar lymph nodes by continuous slice. The black arrow indicates IgA and CD11c gathering area. The scale bar is shown in the lower right corner of each image. FSC, forward scatter; SSC, side scatter.

Next, we detected the distribution of CD11c in the hilar lymph nodes and lungs, where the highest expression levels of IgA presented. The continuous slice of tissues provided the possibility of locating two indicators in one site of two different tissue slices. We found strong signals of CD11c, where lots of IgA presented, especially in the centrilobular nodules (Fig. 4A), the peritrabecular sinuses, and around the trabecula of the lymph nodes (Fig. 4B), which indicated that CD11c-positive immune cells might play an important role in IgA response to M. hyopneumoniae infection. After observation of the histomorphology and tissue localization of the positive cells, it was found that most CD11c-positive cells were DCs and macrophages (Mφ).

### M. hyopneumoniae promotes secretion of IgA in the mouse LDC/B cell coculture model.

**FIG 5:**
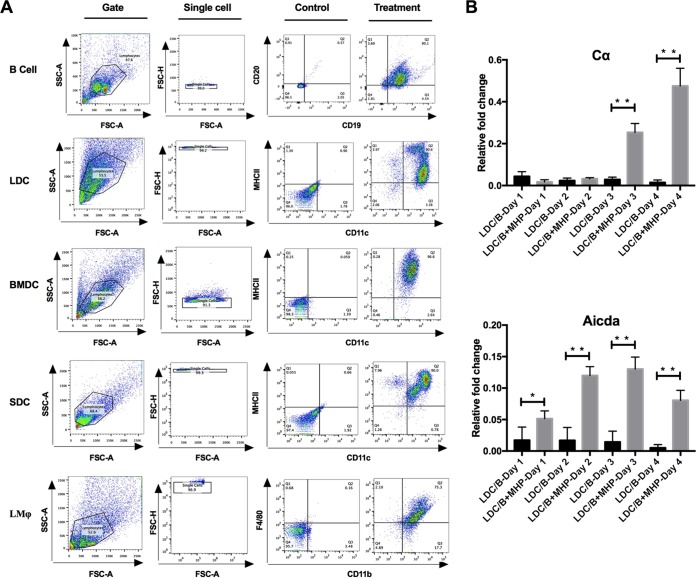
Flow cytometry analysis of mouse cells used in cell coculture model and the transcription levels of IgA CSR-related genes. (A) Flow cytometry analysis of CD19^+^ CD20^+^ B cells, CD11c^+^ MHC-II^+^ lung DCs (LDCs), CD11c^+^ MHC-II^+^ bone marrow-derived DCs (BMDCs), CD11c^+^ MHC-II^+^ spleen DCs (SDCs), and F4/80^+^ CD11b^+^ lung Mφ (LMφ). (B) Expression of Cα and *Aicda* in mouse LDC/B cells and the M. hyopneumoniae whole-cell lysate-stimulated mouse LDC/B cells. Quantitative reverse transcription-PCR (qRT-PCR) was performed to detect the expression of Cα and *Aicda* after coculture for 1, 2, 3, and 4 days, and data were normalized to glyceraldehyde-3-phosphate dehydrogenase (GAPDH) and expressed as a relative fold change. MHP represents that 10 μg/ml M. hyopneumoniae whole-cell lysate was included in the cell culture medium. All experiments were repeated three times independently, and two-tailed *t* test analysis was performed to detect significant differences between the stimulated and nonstimulated groups. *, *P* < 0.05; **, *P* < 0.01.

**FIG 6:**
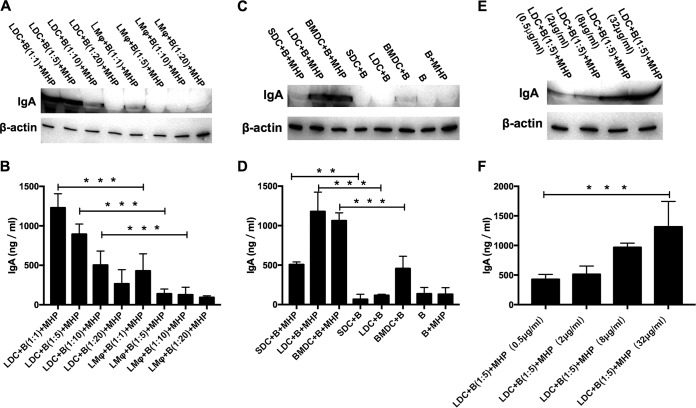
M. hyopneumoniae promotes cell coculture models to secrete IgA. (A and B) Mouse B cells were cocultured with mouse lung DCs (LDCs) or lung Mφ (LMφ) at different ratios, and IgA levels in the culture supernatants were analyzed by Western blotting (A) and ELISA (B) after stimulation for 6 days with 10 μg/ml M. hyopneumoniae whole-cell lysate. All experiments were repeated three times independently, and two-tailed *t* tests were performed to analyze the significance differences between the LDC/B cell and LMφ/B cell-cocultured groups. ***, *P* < 0.001. (C and D) Mouse B cells were cocultured with mouse SDCs, LDCs, or BMDCs at a ratio of 5:1, and IgA levels in the culture supernatants were analyzed by Western blotting (C) and ELISA (D) after coculture for 6 days. All experiments were repeated three times independently, and two-tailed *t* tests were performed to analyze significant differences between the M. hyopneumoniae whole-cell lysate-stimulated group and the nonstimulated group. ***, *P* < 0.001. (E and F) LDCs and B cells were cocultured at a ratio of 1:5, and IgA levels in the culture supernatants were detected by Western blotting (E) and ELISA (F) after stimulation for 6 days with different concentrations of M. hyopneumoniae whole-cell lysate. All experiments were repeated three times independently, and two-tailed *t* tests were performed to analyze significant differences between the low-dose stimulation group and the high-dose stimulation group, ***, *P* < 0.001. MHP represents that 10 μg/ml M. hyopneumoniae whole-cell lysate was included in the cell culture medium.

Based on the above-mentioned findings, we reasoned that lung DCs or lung Mφ might dictate IgA class switching of B cells to resist M. hyopneumoniae infection. To prove this hypothesis, we separated mouse CD11c^+^ major histocompatibility complex class II (MHC-II)-positive DCs from the lung DCs (LDCs) (Fig. 5A) and cocultured them with mouse CD19^+^ CD21^+^ B cells of the spleen (Fig. 5A) in the presence of 10 μg/ml M. hyopneumoniae whole-cell lysate. IgA CSR was mediated by activation-induced cytidine deaminase (AID), which is a well-documented mammalian DNA-editing enzyme encoded by the *Aicda* gene. We analyzed expression of the *Aicda* gene and the cAMP-dependent protein kinase catalytic subunit alpha (Cα) at different time points during LDC/B cell coculture with or without the M. hyopneumoniae stimulation. The results showed that the expression of Cα and *Aicda* in B cells was higher with the M. hyopneumoniae stimulation than without the stimulation (Fig. 5B), which indicated that M. hyopneumoniae promoted the CSR of B cells to produce IgA under the help of LDCs, and IL-10 expression was increased during the first 4 days of coculture (Fig. S2). After 6 days of coculture, the cell culture supernatant was collected for IgA detection by enzyme-linked immunosorbent assay (ELISA) and Western blot analysis. The content of IgA increased with increasing numbers of LDCs. In contrast, mouse CD64^+^ CD11b^+^ lung macrophages (LMφ, Fig. 5A) were poor at inducing secretory IgA (Fig. 6A and B). LDCs played a more important role than LMφ in IgA immune responses to M. hyopneumoniae infection.

Then, we separated CD11c^+^ MHC-II^+^ DCs (Fig. 5A) from different tissues of mice, i.e., the lungs (LDCs), spleen (SDCs), and bone marrow-derived cells (BMDCs) stimulated by IL-4 and granulocyte-macrophage colony-stimulating factor (GM-CSF), and cocultured them with CD19^+^ CD21^+^ B cells of the spleen along with an M. hyopneumoniae whole-cell lysate. Under stimulation, the three types of DCs all promoted B cells to secrete IgA, but SDCs played a relatively weak role in promoting IgA, while LDCs had the strongest promoting effect, and BMDCs had a certain promotion effect without stimulation (Fig. 6C and D). Based on the above-mentioned results, and considering that M. hyopneumoniae is a respiratory pathogen, we selected the model of LDC/B cell coculture to study the mechanism of IgA increase caused by M. hyopneumoniae infection. Our data also showed that the most highly matured BMDCs and low matured lung-derived DCs had the same ability of promoting the secretion of IgA when stimulated by M. hyopneumoniae. However, SDCs, second only to BMDCs in maturity, have a weak ability to stimulate IgA production, which indicated that the ability of DCs to promote IgA CSR might not be related to maturity (Fig. S3).

To detect the ability of M. hyopneumoniae whole-cell lysate to promote the production of IgA in the cell model, we stimulated the cell model with different concentrations of lysate. After 6 days, the amount of IgA was detected. It was found that IgA increased with increased concentration of lysate (Fig. 6E and F), which indicated that M. hyopneumoniae could effectively promote the secretion of IgA in the mouse cell model and was dose dependent.

### M. hyopneumoniae promotes the mouse LDC/B cell coculture model to secrete IgA via TLR2 and TLR4.

**FIG 7:**
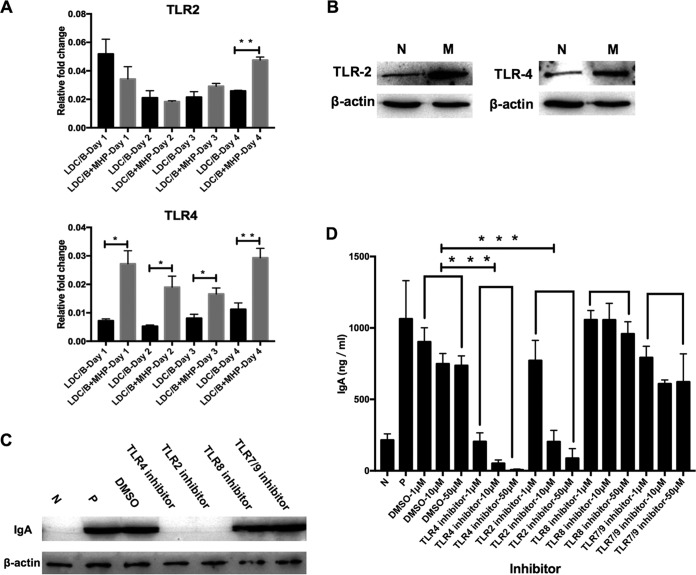
M. hyopneumoniae promotes IgA secretion in LDC/B cell coculture model via TLR2 and TLR4. (A) Expression of TLR2 and TLR4 was detected by qRT-PCR of the mRNA isolated from mouse LDC/B cells cocultured with or without M. hyopneumoniae whole-cell lysate for 1, 2, 3, and 4 days. Data were normalized to GAPDH and expressed as a relative fold change. MHP represents that 10 μg/ml M. hyopneumoniae whole-cell lysate was included in the cell culture medium. All experiments were repeated three times independently and two-tailed *t* tests were performed to analyze significant differences between the stimulated and nonstimulated groups. *, *P* < 0.05; **, *P* < 0.01. (B) TLR2 and TLR4 expression in the mouse LDC/B cells was analyzed by Western blotting after coculture for 6 days with 10 μg/ml M. hyopneumoniae whole-cell lysate (stimulated group, M) or without M. hyopneumoniae stimulation (nonstimulated group, N). β-Actin was used as a reference protein. (C and D) Mouse LDCs and B cells were cultured together for 6 days in the medium with 10 μg/ml M. hyopneumoniae whole-cell lysate and different concentrations (1, 10, or 50 μM) of inhibitors of TLR4, TLR2, TLR8, TLR7/9, or DMSO. N represents coculture of only LDCs and B cells, and P represents LDCs and B cells cocultured with 10 μg/ml M. hyopneumoniae whole-cell lysate. IgA levels in the supernatants with 10 μM inhibitors were analyzed by Western blotting (C), and IgA levels in all groups were measured by ELISA (D). All experiments were repeated three times independently and two-tailed *t* tests were performed to analyze significant differences between the DMSO group and the TLR4 or TLR2 inhibitor group. ***, *P* < 0.001.

From the previous experiments, we knew that M. hyopneumoniae whole-cell lysate could effectively promote the production of IgA by the cell model. To investigate the mechanism behind this phenomenon, we used the mRNA isolated from mouse LDC/B cells of the unstimulated group and the stimulated group after coculturing for 1, 2, 3, and 4 days to detect the expression of TLR2, TLR3, TLR4, TLR6, TLR7, TLR8, and TLR9. The results showed that TLR2 was highly expressed after 4 days, and TLR4 was highly expressed from 1 to 4 days of coculture (Fig. 7A), while TLR8 was downregulated in expression after 3 and 4 days, and TLR9 was downregulated after 4 days. There was no significant difference in the expression of TLR3, TLR6, or TLR7 over this time course (Fig. S4). Then, we used Western blot analysis to detect the protein expression of TLR2, TLR3, TLR4, TLR6, TLR7, TLR8, and TLR9 after 6 days of coculture of the unstimulated and stimulated groups. TLR2 and TLR4 were increased in expression in the stimulated group compared with the unstimulated group (Fig. 7B). TLR6 was increased in the stimulated group, while there was no significant difference in the expression of TLR3, TLR7, TLR8, or TLR9 (Fig. S5). To detect the effect of TLRs on the M. hyopneumoniae-induced secretion of IgA by the cell model, we employed inhibitors of TLR4, TLR2, TLR8, and TLR7/9 in the mouse LDC/B cell coculture model stimulated with 10 μg/ml M. hyopneumoniae whole-cell lysate. After 6 days of coculture, the cell culture supernatant was collected for IgA detection by ELISA and Western blot analysis. The results showed that the inhibitors of TLR4 and TLR2 suppressed the production of IgA, but the TLR8 and TLR7/9 inhibitors did not affect the production of IgA (Fig. 7C and D). This implied that M. hyopneumoniae stimulates an IgA immune response through TLR2 and TLR4.

## DISCUSSION

In this study, we confirmed that M. hyopneumoniae infection will promote a mucosal immune response and found that IgA and CD11c have significant colocalization in lung lesions and hilar lymph nodes. In our previous study, primary porcine B cells and DCs were isolated and cultured *in vitro*, and two times more IgA was excreted in the DC/B cell coculture system than with B cells alone induced by M. hyopneumoniae, suggesting that the coculture of primary porcine DC and B cells can effectively produce IgA under the stimulation of M. hyopneumoniae (34) and indicating that the DC/B cell culture *in vitro* could be a good model for studying the IgA response to M. hyopneumoniae. However, further study about the molecular mechanism of IgA responses was hampered due to the difficulty of culturing the primary porcine cells consistently because of the difference between pigs. Moreover, the poverty of the specific reagents for pigs such as antibodies and inhibitors also hindered the study in depth. A mouse DC and B cell coculture model has been used in the studies about the infection and immunity of Mycoplasma pneumoniae (35, 36). To prove that the mouse DC and B cell coculture model could be also used for the mechanism study about IgA immune response to M. hyopneumoniae, we cocultured mouse DC and B cells and tested IgA response to the stimulation by M. hyopneumoniae whole-cell lysate, which was chosen from the perspective of immunity rather than infection study. The results showed that M. hyopneumoniae promotes the mouse lung DC/B cell coculture model to produce IgA compared to mouse B cells alone, similar to the effect in the porcine DC/B cell coculture system by live microbes of M. hyopneumoniae. These results implied that the mouse lung DC/B cell coculture model stimulated by M. hyopneumoniae cell lysate could reasonably be used for the study of the molecular pathways involved in the IgA immune response.

IgA CSR is the process whereby B cells acquire the expression of IgA. It is a complex process regulated by many factors, including cytokines, endoplasmic reticulum (ER) stress, and other immune cells (37, 38). DCs are potent professional antigen-presenting cells that sensitize B cells to switch on appropriate immune responses to antigen danger signals (39). DCs modulate B cell functions and, consequently, immune responses in various ways. Activated DCs show higher production of IL-6, IL-10, IL-12, TGF-β1, and alpha interferon (IFN-α) (22), and DCs can also highly express BAFF and APRIL, which serve in the generation and maintenance of mature B lymphocytes (40, 41). To further study the main factors involved in DC function, we used DCs from different sources to generate cell models, and with reference to other studies (17, 21–23), the DCs, B cells, and stimulators were cocultured; then, we studied IgA changing under the stimulation of M. hyopneumoniae. Our data showed that the mature cultured BMDCs had the highest maturity, while the lung-derived DCs had low maturity, and the abilities of these DCs to promote the secretion of IgA under stimulation by M. hyopneumoniae were equal. This indicated that the ability of DCs to promote IgA CSR might not be related to maturity. In other words, the surface molecular markers CD80 and CD86 possessed by mature DCs have no significance in promoting the IgA CSR. Although our experiments demonstrate that DC is necessary for M. hyopneumoniae to promote IgA secretion in the *in vitro* cell model, the specific function of DC in this process remains to be further studied, such as with a change in culture method to study if M. hyopneumoniae-specific stimulated DCs can promote the secretion of IgA.

As an important antibody component in mucosal tissues, IgA can neutralize microbial toxins with high affinity and block the adhesion and infection of pathogens through its low-affinity binding system so as to prevent bacteria from damaging the mucosal surface (42), while the first step of M. hyopneumoniae infection is adhesion (43). The complement system plays important and complicated roles during the process of *Mycoplasma* infection and the host’s immune responses (44–46). In one role, the complement system activated by infection can kill *Mycoplasma* spp. by the formation of an attacking membrane complex. In another role, some inflammation-related complement components can promote pathogenicity (47, 48), and IgA has also been found to be related to nephropathy by activating the complement system through lectins and alternative pathways (49).

It is known that LPS stimulates B cells to enhance their antigen-presenting capacity, which is accompanied by the secretion of large amounts of LPS-neutralizing antibodies, and higher expression of TGF-β1 is detected on the surface of LPS-activated B cells (50). The lack of LPS in *Mycoplasma* spp. means that this organism stimulates multiple, diverse effects on the cells of the immune system, including polyclonal activation of B and T cells (51). *Mycoplasma*-derived macrophage-activating lipopeptide 2 (MALP-2) and triacylated lipoproteins derived from M. pneumoniae resulted in potent enhancement of humoral and cellular antigen-specific immune responses at both the systemic and mucosal levels (52). In the present study, M. hyopneumoniae cell lysate promoted the production of secretory IgA antibody by the DC/B cell coculture model. This indicated that M. hyopneumoniae may possess an immunogen like MALP-2 or triacylated lipoproteins which, like LPS, can promote the immune response of B cells from different species. Moreover, microbial TLRs can also initiate germ line Cα gene transcription and CSR from Cμ to Cα in mouse B cells; however, the mechanism by which TLRs trigger IgA CSR in B cells remains unclear (53). Studies have shown that capsular structures exist in M. hyopneumoniae and other *Mycoplasma* spp. (54, 55). The capsular polysaccharide can promote the immune response of the body through the TLR4 pathway (56). Studies have shown that the capsular component of Mycoplasma pneumoniae can promote the secretion of IL-10 by binding to DCs (57), which is consistent with the experimental results of the increase in IL-10 in the cell coculture model of our research. In addition, increasingly more studies have shown that some other pathogen-associated molecular patterns (PAMPs) can promote immune responses through the TLR4, such as heat shock protein 60 (Hsp60) (58, 59), and Hsp60 exists in M. hyopneumoniae; another study shows that Hsp60 has good immunogenicity in the first natural infection of M. hyopneumoniae (60), which suggests that the capsule or Hsp60 of M. hyopneumoniae may play an important role in promoting the secretion of IgA.

Although there are many available vaccines against M. hyopneumoniae on the market, a large number of cases of M. hyopneumoniae are still reported (61, 62). Therefore, our understanding of the mechanism of protective immunity is crucial in reducing the transmission of M. hyopneumoniae infection. Our findings indicate that compared with IgG and IgM, IgA significantly increases during M. hyopneumoniae infection. M. hyopneumoniae artificially infected pigs consume a normal diet and appear to present a good mental state, but pathological sections of the lung, trachea, and nasal mucosa present severe pathological changes, which will trigger further lesions and secondary infections. It is urgent to develop more effective vaccines for the prevention and treatment of M. hyopneumoniae.

In summary, we present a new cell model for mucosal immunity induced by mycoplasmas. This cell model provides a tool for studying related pathways and developing effective vaccines that can induce secretory IgA.

## MATERIALS AND METHODS

### Animals and ethics statement.

All pigs in this experiment were 15 to 20 days old and were obtained from the Swine Breeding Center in Beijing, China. All pigs were M. hyopneumoniae antibody negative, as tested by an M. hyopneumoniae antibody test kit (Idexx, ME, USA), and were M. hyopneumoniae negative in the nasal swabs, as tested by PCR (Fig. S1A). Pigs were housed in groups at China Agricultural University under hygienic and ethically approved feeding conditions. C57BL/6 mice (B6) were purchased from the Beijing Vital River Laboratory Animal Technology Company. Mice were maintained in specific-pathogen-free facilities at China Agricultural University, and the female mice were used at 4 to 6 weeks of age.

All animal care procedures and experiments were approved by the Beijing Association for Science and Technology and were in compliance with the Beijing Laboratory Animal Welfare and Ethics Guidelines issued by the Beijing Administration Committee of Laboratory Animals. All animal studies were performed in accordance with the China Agricultural University Institutional Animal Care and Use Committee guidelines (CAU20180106-2) approved by the Animal Welfare Committee of China Agricultural University.

### Bacterial strains and culture methods.

M. hyopneumoniae strain RM48 was kindly donated by Qingchun Shen. The liquid medium for the culture of M. hyopneumoniae was prepared by dissolving 1.56 g of M. hyopneumoniae medium (QingDao Hopebio Biotech, Beijing, China) with 75 ml of double-distilled water and autoclaving at 121°C for 15 min; then, 25 ml inactivated pig serum and 1 mM ampicillin were added to the medium after it was cooled to room temperature. M. hyopneumoniae was cultured for 5 days in a 37°C and 5% CO_2_ incubator, and a color-changing unit (CCU) assay was used for counting live bacteria. M. hyopneumoniae was centrifuged for 30 min at 12,000 rpm and diluted to 10^8^ CCU/ml with phosphate-buffered saline (PBS) for the use of challenge in pigs. M. hyopneumoniae precipitation solution was washed three times with PBS, passed through a 0.1-μm filter after ultrasonic lysis, and then diluted to a final concentration of 5 mg/ml with PBS as M. hyopneumoniae whole-cell lysate.

### Animal infection experiment.

Eight pigs were randomly divided into two groups and housed separately. Four pigs (numbers 5, 6, 7, and 8) were challenged intratracheally with 3 ml and intranasally with 2 ml of M. hyopneumoniae (10^8^ CCU/ml). The other four pigs were inoculated with PBS via the same route (numbers 1, 2, 3, and 4). Nasal swabs and peripheral blood were collected every 5 days. IgA, IgG, and IgM were detected by ELISA. After 20 days, necropsy examinations were performed, and the lung lesions were scored according to the assessment method described by Ostanello et al. (63). In brief, the lesion area in the left heart lobe, right heart lobe, left tip lobe, right tip lobe, right diaphragmatic lobe, left diaphragmatic lobe, and middle lobe were assessed and scored, with 0 indicating no lesions, 1 indicating lesions of an area less than 25% of the surface, 2 indicating lesions of an area between 25 and 50% of the surface, 3 indicating lesion areas between 50 and 75% of the surface, and 4 indicating lesion areas equal to or more than 75% of the surface. About 300 ml BALF was collected aseptically from the lungs, and turbinate bone, trachea, hilar lymph node, and lung tissues were collected aseptically for hematoxylin and eosin and immunohistochemistry.

### Histological examination of pig respiratory tissues by hematoxylin and eosin staining and immunohistochemistry method.

Porcine nasal tissue, turbinate, hilar lymph node, and lung tissues were preserved in 4% paraformaldehyde solution, embedded in paraffin, sectioned at 6 μm, mounted onto positively charged glass slides, and stained with hematoxylin and eosin (H&E) according to the standard protocol used in the Department of Pathology at China Agricultural University.

For the immunohistochemistry staining of IgA and CD11c, tissue samples were preserved in 4% paraformaldehyde solution, dehydrated and embedded in paraffin following routine methods, sectioned at 6 μm, and mounted onto positively charged glass slides. After rinsing three times (5 min per time) with 0.01 M PBS (pH 7.4), 0.02% KH_2_PO_4_, 0.29% N_2_HPO_4_, 0.02% KCl, 0.8% NaCl, 0.05% bovine serum albumin (BSA), 0.05% Tween 20, and 0.0015% Triton X-100, each paraffin section was blocked with 100 to 400 μl of blocking solution for 1 h. After removing the blocking solution, 100 to 400 μl of primary antibody diluted in blocking solution was added and incubated at 4°C for 2 h. Then, 3,3′ diaminobenzidine (DAB) detection was performed according to the manufacturer’s instructions. For IgA detection, the antigen was repaired for 5 min and incubated with goat anti-pig IgA antibody (polyclonal; Bethyl, TX, USA) after being diluted 1:200. For CD11c detection, the antigen was repaired for 3 min and incubated with rabbit anti-pig CD11c antibody (polyclonal; ABclonal, Wuhan, China) after being diluted 1:200.

### Preparations of mouse mononuclear cells from lungs, spleen, and bone marrow.

Mice were euthanized, and the lungs, spleen, and bone marrow were taken. The isolation of lung mononuclear cells was performed as previously described (64, 65). In brief, the pulmonary circulation was perfused with saline to remove the intravascular pool of cells after euthanization. The lungs were carefully separated from the thymic and cardiovascular remnants, thoroughly minced using iridectomy scissors, and incubated for 60 min in digestion medium containing type I collagenase (Gibco, Grand Island, NY, USA) in a humidified incubator at 37°C and 5% CO_2_. Then, 10 mM EDTA was added and incubated for 10 min. Tissue fragments were disrupted mechanically by pipetting and filtered with a 70-μm cell strainer. The mononuclear cells in the filtrate were centrifuged and washed twice with RPMI 1640 medium (Gibco) before isolation of LDCs and LMφ in the subsequent experiments. The spleen was gently crushed with a piston on the nylon screen, and mononuclear cells were harvested in RPMI 1640 medium. Bone marrow DCs (BMDCs) were generated from single-cell bone marrow suspensions prepared from mouse femurs and tibias (66). Bone marrow cells were cultured at a density of 1 × 10^6^ cells/ml in 24-well plates in RPMI 1640 medium supplemented with 10% fetal bovine serum (FBS), streptomycin, and penicillin in the presence of 25 ng/ml recombinant mouse granulocyte-monocyte colony-stimulating factor and 10 ng/ml IL-4 (PeproTech, Rocky Hill, NJ, USA). The culture medium was renewed on days 2, 4, and 6. On day 7, BMDC clusters were harvested, washed, and enriched.

### Isolation of B cells, DCs, and LMφ.

B cells were isolated from mouse spleens using anti-CD19 magnetic beads according to the manufacturer’s instructions. In brief, the cells were incubated with anti-CD19 magnetic beads for 15 min on ice and washed with 1 ml of MACS buffer (solution containing phosphate-buffered saline [PBS], pH 7.2, 0.5% bovine serum albumin [BSA], and 2 mM EDTA) (Miltenyi Biotec, Bergisch Gladbach, Germany). The cellular suspensions collected were washed twice in MACS buffer and passed through a magnetic column. Then, CD19^+^ B cells were isolated by positive selection, washed, resuspended in complete RPMI 1640 medium, and counted before coculture with DCs or lung macrophages (LMφ), as described below.

From the mononuclear cells isolated from the mouse lungs, spleen, and bone marrow, CD11c^+^ DCs were isolated using CD11c magnetic beads (Miltenyi Biotec, Bergisch Gladbach, Germany). CD64^+^ LMφ were isolated using a CD64 primary antibody (clone X54-5/7.1; BioLegend, CA, USA) and anti-mouse IgG microbeads (Miltenyi Biotec), as described for B cell isolation.

### *In vitro* cell cocultures.

The culture medium used for cell culture was RPMI 1640 medium supplemented with 10% FBS, streptomycin, penicillin, and 2 mM l-glutamine. B cells were added to round-bottom microtest wells at 10^5^ cells/well and mixed with isolated DCs or LMφ at different cell ratios. For the M. hyopneumoniae stimulation assays, the coculture cells were incubated with M. hyopneumoniae whole-cell lysate. For the TLR inhibition assays, we used 1, 10, and 50 μM inhibitors of TLR4 (MCE, NJ, USA) (67–71), TLR2 (MCE) (72), TLR8 (MCE) (73), and TLR7/9 (MCE) (74, 75) dissolved in dimethyl sulfoxide (DMSO) (10 mM). The DMSO control was set up at the same time.

### ELISA.

The contents of IgA, IgG, and IgM in the peripheral blood serum and nasal swabs of pigs were detected with a pig IgA ELISA quantitation kit (Bethyl, TX, USA), pig IgG ELISA quantitation kit (Bethyl), and pig IgM ELISA quantitation kit (Bethyl). The content of secretory IgA in the cell coculture model was determined using a mouse IgA ELISA quantitation kit (Bethyl). ELISA was performed according to the manufacturer’s instructions. The data were analyzed using the ELISA Calc software.

### Western blot analysis of the Toll-like receptors’ expression.

After 6 days of cell coculture, the cells were collected and lysed with protein lysate (Thermo Fisher Scientific, MA, USA) and then quantified, and equal amounts of sample proteins were resolved by 12% SDS-PAGE gels and transferred onto nitrocellulose membranes (Bio-Rad, CA, USA). Then, membranes were incubated in 5% skim milk in PBST (PBS containing 0.5‰ Tween 20) for 2 h, followed by incubation for 2 h with the primary antibodies anti-mouse TLR2 (polyclonal; Sigma, St. Louis, MO, USA), anti-mouse TLR4 (clone 76B357.1; Abcam, Cambridge, UK), anti-mouse TLR6 (polyclonal; Abcam), anti-mouse TLR7 [clone EPR2088(2); Abcam], anti-mouse TLR8 (polyclonal; ABclonal, Wuhan, China), and anti-mouse TLR9 (polyclonal; ABclonal). After the membranes were washed three times with PBST, the primary antibody was detected by incubating with the secondary antibody horseradish peroxidase (HRP)-conjugated rabbit anti-mouse IgG (H+L; ABclonal). The membranes were washed three times with PBST, and the antibody was detected using the *EasySee* Western blot kit (TransGen Biotech, Beijing, China).

### Flow cytometry analysis.

Cells were isolated as described above. Before staining, cells were washed and resuspended in staining buffer containing 1× PBS and 1% FBS. To block nonspecific staining, an anti-CD16/32 antibody (clone 93; BioLegend, CA, USA) was added. Then, antibodies against cell surface markers were added, and cells were incubated with cells for 25 min at 4°C. After staining, the cells were washed twice and analyzed immediately. Flow cytometry data were analyzed using the FlowJo software. The antibodies used in flow cytometry analysis included allophycocyanin (APC) anti-mouse CD11c antibody (clone N418), phycoerythrin (PE) anti-mouse MHC-II antibody (clone M5/114.15.2), Brilliant Violet 421 anti-mouse CD80 antibody (clone 16-10A1), Brilliant Violet 421 anti-mouse CD86 antibody (clone GL-1), PE anti-mouse F4/80 antibody (clone BM8), fluorescein isothiocyanate (FITC) anti-mouse CD19 antibody (clone MB19-1), and peridinin chlorophyll protein (PerCP) anti-mouse IgG (clone Poly4053) and were from BioLegend. FITC anti-mouse CD11b (clone M1/70) and anti-mouse CD20 (clone L26) were from Abcam (Cambridge, UK).

### PCR and quantitative PCR tests.

Total RNA was extracted using an RNAprep pure cell kit (Tiangen Biotech, Beijing, China) and used for reverse transcription with Maxima H minus cDNA synthesis master mix with double-stranded DNase (dsDNase) (Thermo Fisher Scientific, MA, USA). All procedures followed the manufacturer’s instructions. Then, 50 ng of cDNA was used to perform qualitative PCR analysis of specific genes using the primers described in Table S1 in the supplemental material. PCR amplification products were analyzed by 2% agarose gels, and PCR bands were observed under a UV illuminator. Images of the gels were recorded for densitometric analyses. Quantitative RT-PCR (LightCycler 96; Roche, Basel, Switzerland) was performed using 25 ng of cDNA. The Maxima SYBR green kit (TransGen Biotech, Beijing, China) was used to prepare the master mix. The PCR profile was set using the LightCycler 96 software, as follows: initial denaturation at 95°C for 5 min, 40 cycles of 95°C, 55°C, and 72°C, followed by elongation at 72°C and termination at 20°C. The transcription level of genes was normalized to the GAPDH housekeeping gene in the same tissue samples.

PCR was performed as follows: initial denaturation at 95°C for 5 min, 35 cycles of 95°C, 50°C, and 72°C, followed by elongation at 72°C and termination at 20°C. The primers used are described in Table S1 in the supplemental material, and the gene used in the PCRs for detecting M. hyopneumoniae was a gene encoding a conserved hypothetical protein and is named *mhp165* (GenBank accession no. AE017332, M. hyopneumoniae strain 232 complete genome; bp 195124 to 201267). We compared the *mhp165* gene sequences of six M. hyopneumoniae strains (GenBank accession numbers AE017244, AE017243, EU658732, EU658731, EU658730, and CP002274). The consensus sequence among the M. hyopneumoniae strains was determined after the alignment analysis. In addition, similar sequences have not been found in other *Mycoplasma* species and bacterial strains by MEGA-BLAST or BLASTN in the NCBI (33). The target gene fragments we chose in this paper were bp 199131 to 199370 (MHP240) and bp 199034 to 199198 (MHP165).

### Statistical analysis.

Two-tailed *t* tests were performed to detect the significance between the study groups which fulfilled the criteria of normality (performed by Shapiro-Wilk normality test), and a nonparametric Mann-Whitney test was used in case those data did not fulfill the criteria of normality. The results were expressed as the mean ± standard error of the mean (SEM) for the obtained values. For analysis, the probability value (*P*) was considered statistically significant at *, *P* < 0.05; **, *P* < 0.01; ***, *P* < 0.001; and ****, *P* < 0.0001. All experiments were repeated three times independently, and statistical analyses were performed using GraphPad Prism version 5.00 for Windows and the GraphPad software.

## Supplementary Material

Supplemental file 1
