# Supplementary material for: Toll-Like Receptor 2 (TLR2) and TLR4 Mediate the IgA Immune Response Induced by Mycoplasma hyopneumoniae
Source: Infect Immun. 2019 Dec 17;88(1):e00697-19. doi: 10.1128/IAI.00697-19 (PMC6921651; doi:10.1128/IAI.00697-19)
Supplement: Supplemental file 1 [file IAI.00697-19-s0001.pdf]

TLR2 and TLR4 mediate IgA immune response induced by *Mycoplasma hyopneumoniae*

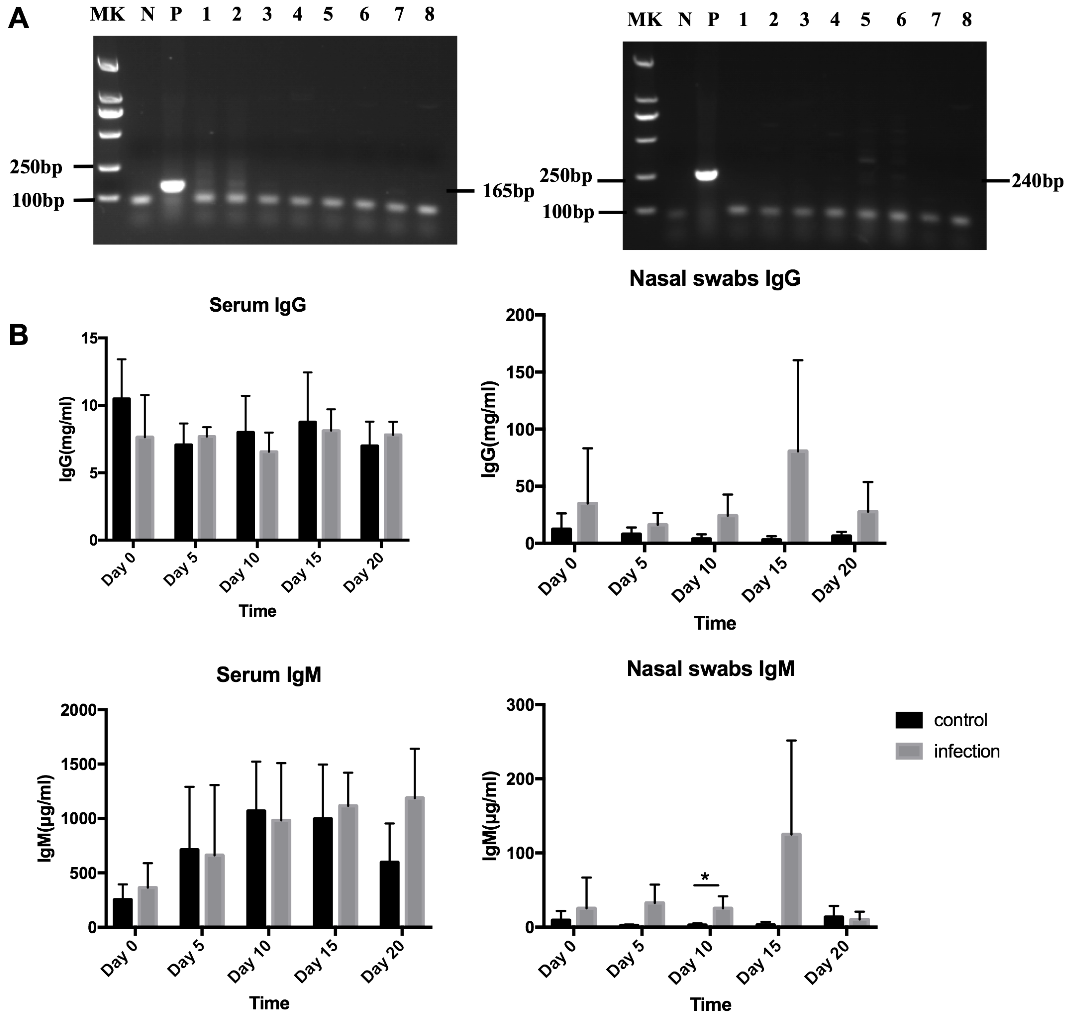

Figure S1. Identifications of *M. hyopneumoniae* by PCR and detections of the IgG and IgM levels in *M. hyopneumoniae*-infected and control pigs. **(A)** Detection of *M. hyopneumoniae* conserved genes in the nasal swabs of the infection and control groups by PCR. The genes used in the PCR reactions for *M. hyopneumoniae* detecting was a conserved hypothetical protein named mhp165 (GenBank accession no. AE017332, *M. hyopneumoniae* strain 232 complete genome; bp 195124– 201267), the target gene fragment we choose in this manuscript were 199034–199198 (bp) (MHP165; Figure S1 A left) and 199131–199370 (bp) (MHP240; Figure S1 A right). MK: DNA marker, N: PCR negative control, P: *M. hyopneumoniae* PCR positive control. 1–8: the pig lung samples. **(B)**. Serum IgG, IgM and nasal swabs IgG, IgM of infection and control groups were detected by ELISA. T tests of two-tailed analysis were performed to detect the significance between the infected group and the control

group of serum IgG, nasal swabs IgG and serum IgM, and there was no significant difference between them. Mann Whitney test were performed to detect the significance between the infected group and the control group of nasal swabs IgM, * represent P < 0.05.

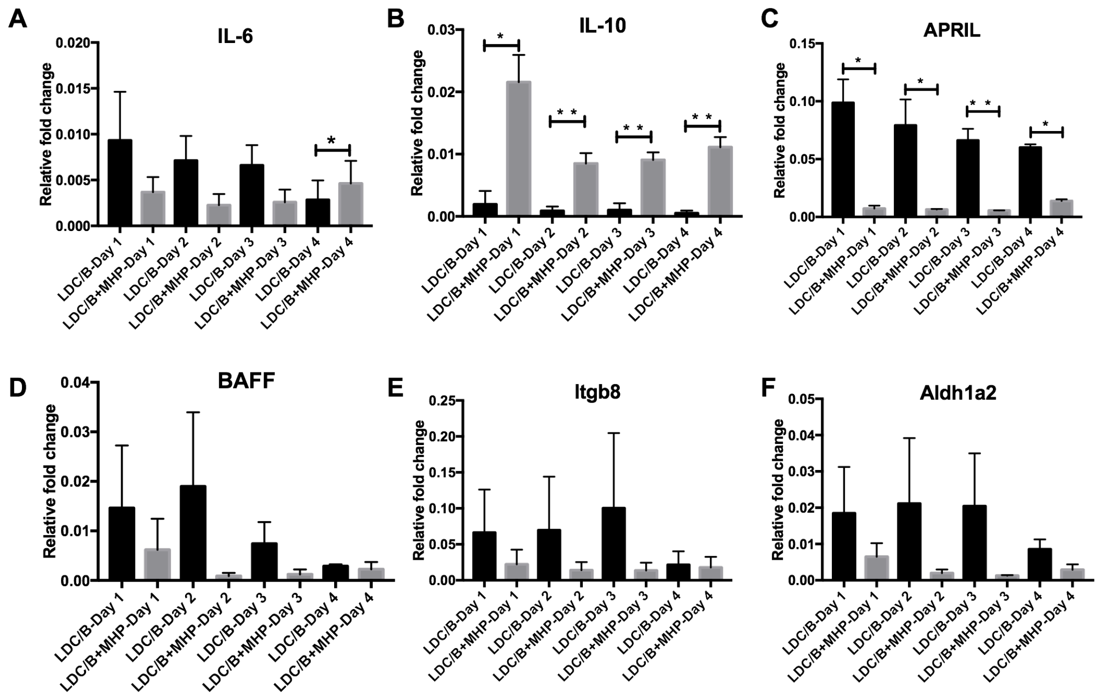

Figure S2. qRT-PCR of mRNA isolated from LDC/B cells in the control group and the stimulated group after co-cultured for 1, 2, 3 and 4 days. IL-6 **(A)**, IL-10 **(B)**, APRIL **(C)**, BAFF **(D)**, Itgb8 **(E)** and Aldh1a2 **(F)** were detected at the transcriptional level. Data were normalized to GAPDH and expressed as a relative fold-change. MHP represents that 10 µg/ml *M. hyopneumoniae* whole cell lysate was included in the cell culture medium. All experiments were repeated three times independently and T tests of two-tailed analysis were performed to detect the significance between the unstimulated group and the stimulated group. * represent P < 0.05; **, represent P < 0.01.

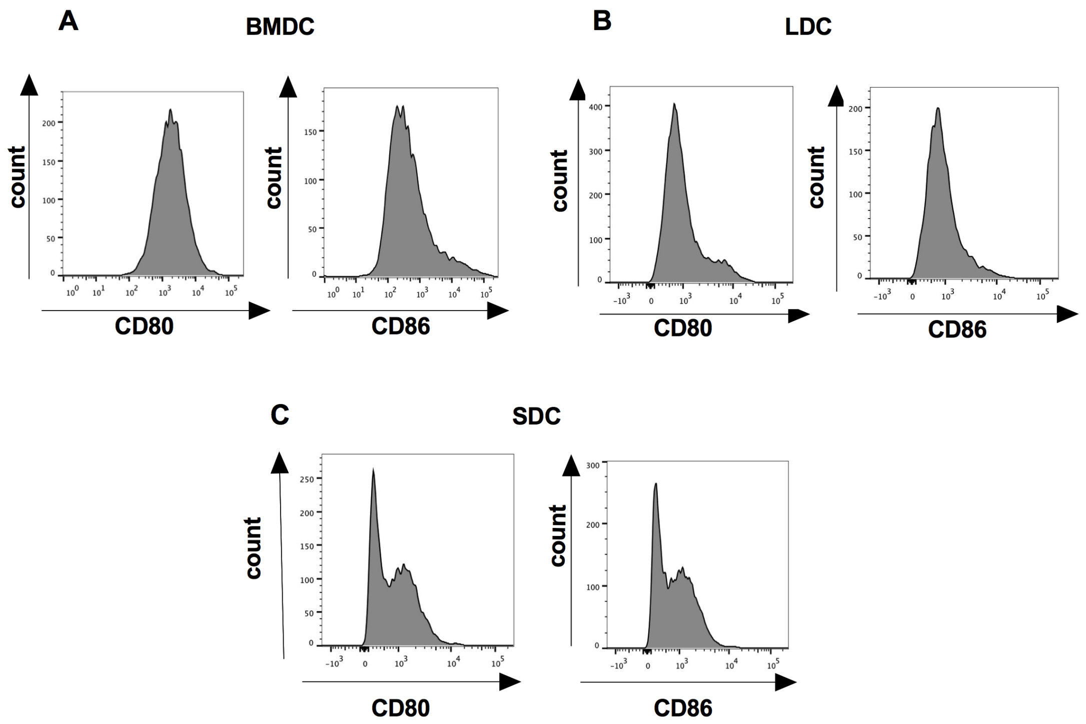

Figure S3. Detection of the maturity of BMDCs, LDCs and SDCs. CD11c$^+$ MHCII$^+$ BMDCs **(A)**, CD11c$^+$ MHCII$^+$ LDCs **(B)** and CD11c$^+$ MHCII$^+$ SDCs **(C)** were collected to detect the positivity to CD80 and CD86.

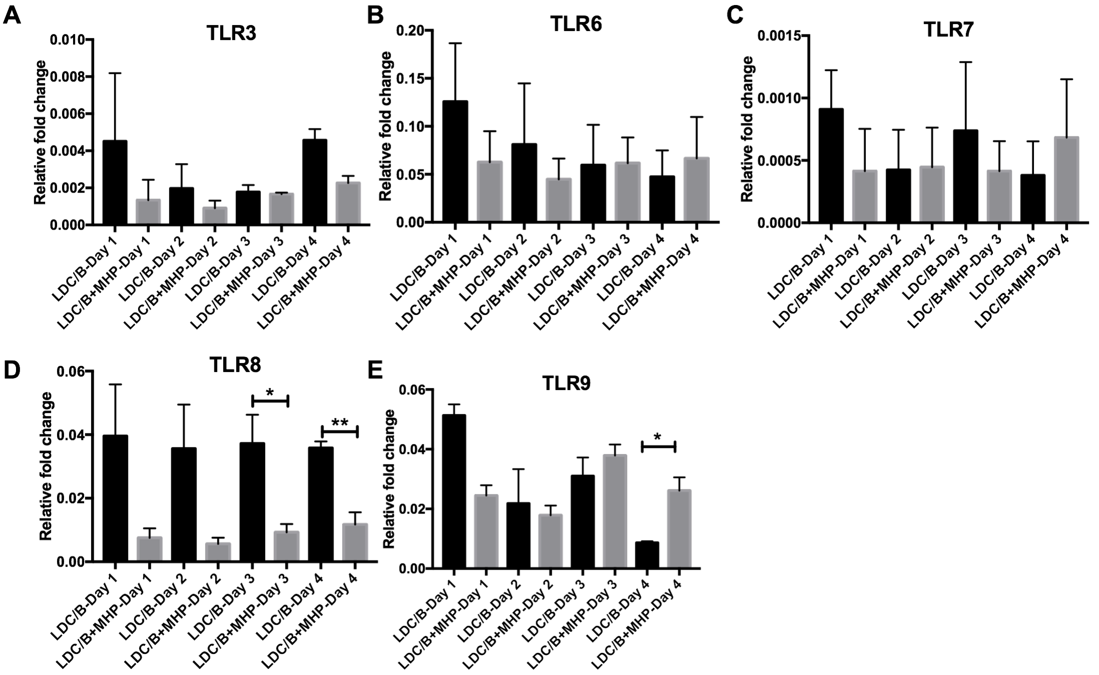

Figure S4. Transcriptional changes of the TLRs in LDC/B cells in the control group and stimulated group. qRT-PCR of mRNA isolated from LDC/B cells of the unstimulated group and stimulated group after co-culture for 1, 2, 3 and 4 days. TLR3 **(A)**, TLR6 **(B)**, TLR7 **(C)**, TLR8 **(D)** and TLR9 **(E)** were detected at the transcriptional level. Data were normalized to GAPDH and are expressed as the relative fold-change. MHP represents that 10 µg/ml *M. hyopneumoniae* whole cell lysate was included in the cell culture medium. All experiments were repeated three times independently and T tests of two-tailed analysis were performed to detect the significance between the unstimulated group and the stimulated group. * represent $P < 0.05$; **, represent $P < 0.01$.

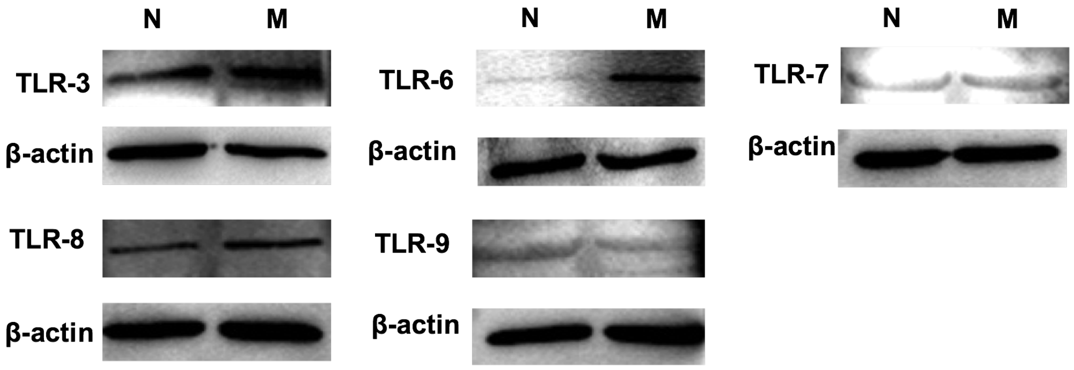

Figure S5. Protein expression changes of the TLRs in LDC/B cells of the control group (N) and stimulated group (M). The stimulation was 10 μg/ml *M. hyopneumoniae* whole cell lysate. Western blot analysis detected TLR3, TLR6, TLR7, TLR8 and TLR9 expression in LDC /B cells after 6 days co-culture of the control group and stimulated group. β-actin was included as a reference protein.

Table 1. The primers used in the article.

**Primer**	**Sequence(5'-3')**	**Primer**	**Sequence(5'-3')**
MHP240F	CAATCCCGGTATTTGTTGAGATCGC	IL6R	GTGCCTCTTTGCTGCTTTCAC
MHP240R	GACTAAAAACCTACCAAACAAAAACAAGGTAG	APRILF	TTGCTCTTTGGTTGAGTTGGGG
MHP165F	TGCCCAGGATATTTCCGATCCAGA	APRILR	GAGTGTTTCTTCTTGTGCTTCT
MHP165R	AGACCTGAAGAACGTGCATGGAGA	TLR2F	CGCTCCAGGTCTTTCACCTC
CαF	CTGATTCACGATTACTTCCCTT	TLR2R	AGGTCACCATGGCCAATGTA
CαR	AGGAGGAGGAGGACCAGAGCACTT	TLR3F	CTGGGTCTGGGAACATTTCT
Aldh1a2F	ACCGTGTTCTCCAACGTCACTGAT	TLR3R	TTGCTGAACTGCGTGATGTA
Aldh1a2R	TGCATTGCGGAGGATACCATGAGA	TLR4F	TCCCTGCATAGAGGTAGTTC
IL10F	GGCCAGAGCCACATGCTCCTAG	TLR4R	ACTCTGGATAGGGTTTCCTG
IL10R	CTTCTCACCCAGGGAATTCA	TLR6F	TTAACTGACCTTCCTGGGTGTGG
AicdaF	GCCACCTTCGCAACAAGTCT	TLR6R	GCAGAACAGTATCACAGGACAGTGG
AicdaR	CCGGGCACAGTCATAGCAC	TLR7F	ATACCTGGCCACTGATGTGA
BAFFF	AATGCACAGATTTCACGCAACG	TLR7R	ATACCTGGCCACTGATGTGA
BAFFR	GCGGGCTCCGTTTCTCATAACT	TLR8F	GTTATGTTGGCTGCTCTGGTTCAC
Itgb8F	AGTGGCCGAGGTGTTTGCATCT	TLR8R	TCACTCTCTTCAAGGTGGTAGC
Itgb8R	CGCAGGTGCCTCTCCCGCTGCA	TLR9F	GACTTACTGTTGGAGGTGCAGACC
IL6F	GGTACATCCTCGACGGCATCT	TLR9R	GAACACCACGAAGGCATCATAGG
